# Finite Element Modelling for Predicting the Puncture Responses in Papayas

**DOI:** 10.3390/foods10020442

**Published:** 2021-02-18

**Authors:** Nurazwin Zulkifli, Norhashila Hashim, Hazreen Haizi Harith, Mohamad Firdza Mohamad Shukery, Daniel Iroemeha Onwude, Masniza Sairi

**Affiliations:** 1 Department of Biological and Agricultural Engineering, Faculty of Engineering, Universiti Putra Malaysia, Serdang 43400, Malaysia; nurazwinzulkifli@gmail.com (N.Z.); hazreen@upm.edu.my (H.H.H.); firdza@upm.edu.my (M.F.M.S.); 2 SMART Farming Technology Research Centre (SFTRC), Faculty of Engineering, Universiti Putra Malaysia, Serdang 43400, Malaysia; 3 Department of Agricultural and Food Engineering, Faculty of Engineering, University of Uyo, Uyo 52021, Nigeria; daniel.onwude@empa.ch; 4 Malaysian Agricultural Research and Development Institute, Serdang 43400, Malaysia; masniza@mardi.gov.my

**Keywords:** papayas, finite element method, puncture mechanics, yielding behaviour

## Abstract

This study aims to develop a finite element (FE) model to determine the mechanical responses of Exotica papayas during puncture loads. The FE model of the puncture-test was developed using the ANSYS 19.1 software. The proposed framework combined the finite element method and statistical procedure to validate the simulation with the experimental results. Assuming the elastic-plastic behaviour of papaya, the mechanical properties were measured through tensile test and compression test for both skin and flesh. The geometrical models include a quarter solid of papaya that was subjected to a puncture test with a 2 mm diameter flat-end stainless-steel probe inserted into the fruit tissues at 0.5 mm/s, 1 mm/s, 1.5 mm/s, 2 mm/s, and 2.5 mm/s. The FE results showed good agreement with the experimental data, indicating that the proposed approach was reliable. The FE model was best predicted the bioyield force with the highest relative error of 14.46%. In conclusion, this study contributes to the usage of FE methods for predicting the puncture responses of any perishable fruit and agricultural products.

## 1. Introduction

Papaya is one of the commercial fruits that is widely planted in most tropical and sub-tropical countries. Yet, the average post-harvest losses reported for papaya in Malaysia is estimated at around 44% each year [1]. Therefore, the availability and accessibility of the papaya must be increased by reducing the number of post-harvest losses. Reduction in these losses would increase the number of fruits available for consumption and thus leads to growing consumer demand. This can be achieved by maintaining the textural quality and improving the shelf-life of the papaya during the handling process, mainly during the distribution from farm to retail.

Papayas are often exposed to deterioration of physical structure caused by rough handling during post-harvest operations. The area or spot of impact can then serve as infection sites for numerous wound pathogens that result in many severe diseases [2]. These spots, even without infection by pathogens, are unsightly and cause moisture loss and excessive shrivelling [3,4]. Besides, the textural property of fruits may decline greatly during the post-harvest supply chain. For instance, exposure to cold temperature reduces the respiration rate and induces the effect of shrinkage of the fruit’s skin [5]. Since papaya is consumed after peeling, not only is its freshness reduced but it also becomes prone to desiccation leading to spoilage.

The evaluation of mechanical properties is important to predict yielding behaviour in fruit. Since fruit is anisotropic, it exhibits a complex hierarchy of biological tissue structures, combining different structural tissues [6]. These tissues are composite materials, combining stiff reinforcing elements and a compliant binding medium that assemble to form the macroscopic tissue structure (Fanta et al. 2013). The mechanical responses of fruit towards external and internal loading are greatly influenced by the microscopic properties of fruit tissues such as the epidermal of cell space, the thickness of cuticle layer, and the length and width of epidermis cell [7]. A puncture test is usually performed to measure mechanical properties of fruits [8] This instrumental test requires a small sample volume, and the characterization of the multi-layered tissue complex can be evaluated at different depths of insertion [9]. The Magnes–Taylor test is commonly used to assess the firmness and stiffness of fruit, by determining properties such as the contact modulus and rupture force [10].

With advances in numerical method and computer simulation, finite element method (FEM) has been used to solve the mechanical-based problems that fruits encounter during quasi-static and dynamic loadings [11,12]. Several studies have been conducted on FE modelling and simulation analysis of fruits under biaxial tension-compression [13,14] and drop tests [15,16], but still, to this date, no FE-puncture model has been proposed.

Therefore, this study aims to develop a three-dimensional (3D) FE model to simulate the probe-papaya interaction during puncture test using the ANSYS software. The present study focuses on developing an FE model to predict the mechanical response of papaya, with the effect of different puncture velocities. The fruit model was presented as a multibody system, and the obtained mechanical parameters of skin and flesh were used for the identification of parameters of the material model. The method presented exploits some prior knowledge about the characterization of the mechanical properties of papaya, and about defining the contact area between the probe tip and the fruit tissues. This is the first occasion that this numerical method has been investigated for the assessment of papayas for puncture test. The model was validated by comparing the simulation results with experimental data. The results presented will help to provide references on the behaviour of fruits over various type of puncture loads. This is the first occasion where this numerical method has been investigated for predicting the puncture responses in papaya.

## 2. Materials and Methods

### 2.1. Samples Preparation

With reference to the colour chart [17], *Carica papaya* cv. “Exotica” papayas in colour break stage-index two were obtained from a commercial exporter in Pasar Borong Seri Kembangan, Serdang, Selangor, Malaysia. The fruits were randomly selected based on uniformity in size, shape, and their being defect-free and then stored for seven days in a controlled refrigerated cold room at 12 ± 1 °C with 80% to 85% relative humidity. Each fruit was left in ambient temperature for about 2 h prior to the assessment of mechanical and physical properties, which was held at day one, four, and seven.

### 2.2. Instrumental Tests

**Figure 1:**
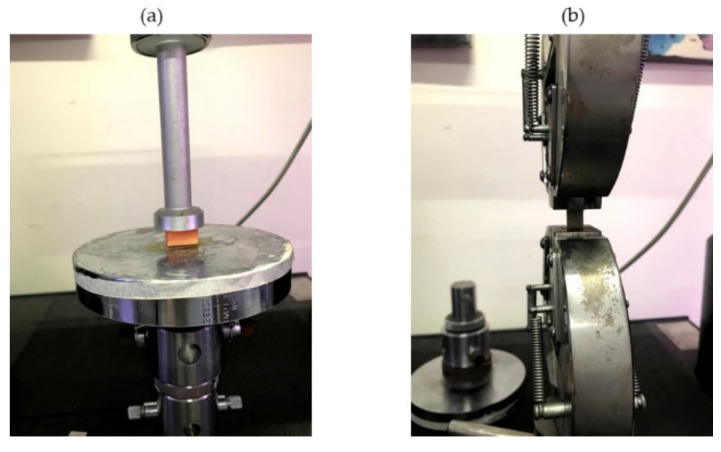
The flesh sample was compressed (**a**); the skin sample was tensioned (**b**).

Based on the existing standard test of food materials [18], mechanical testing was setup to characterize the mechanical properties of both flesh and skin of papaya, to be later used as the initial input parameters for the FE simulations (Figure 1). For the measurement of elastic modulus and failure stress of flesh tissue, the compression test was performed using the universal testing machine (Instron 5543, Instron Corp., Norwood, MA, USA). With the seed and placenta removed, the papaya sample was prepared into 25 mm × 20 mm (length × width) dimensions compressed between two rigid metal plates. The force-deformation data were recorded after being compressed at 60% of compressibility level. Using the same universal testing machine, the tensile testing was performed on the skin to measure the elastic modulus and failure stress. The skin sample with the dimensions of 30 mm × 15 mm in length and width respectively was prepared and the displacement was set to 10 mm. For each storage replication, five samples from five fruits were extracted for the measurement of compression and tensile properties.

A thin layer of green coloured skin was removed, and the uniformly sized cylindrical sample of the papaya flesh was prepared for the measurement of density. For each storage replication, five samples from five fruits were extracted through a smooth-surfaced tube with the uniform size of 20 mm × 25 mm. Each sample was weighted using the electronic balance (AND GF-30K, Tokyo, Japan) with 0.001 g accuracy. With the known weight and dimensions, the density of each sample was calculated based on the ratio of mass to volume.

Using the same fruit for density measurement, five similarly sized cylindrical samples were prepared for the measurement of Poisson’s ratio. The standard procedure of the oven drying method [19] was applied, with cubes heated at 105 °C and 1 atm for 24 h. The differences in the initial weight and the final weight were used to calculate the moisture content.

(1) $$MC=\frac{wt_{1}-wt_{2}}{wt_{1}}\times 100\%$$

where $wt_{1}$ is the initial weight of wet cubes and $wt_{2}$ is the final weight of dried cubes. From the measured *MC*, the calculation for Poisson’s ratio, ν was calculated using the formula proposed by [20], which is given by the following:

(2) $$\nu =\;\frac{0.5MC+0.1\left(100-MC\right)}{100}$$

The constant values of 0.5 and 0.1 indicate the ν value for fresh and dried cube, respectively. The same value of density and ν of flesh sample was considered for the skin sample, respectively.

### 2.3. Puncture Test

**Figure 2:**
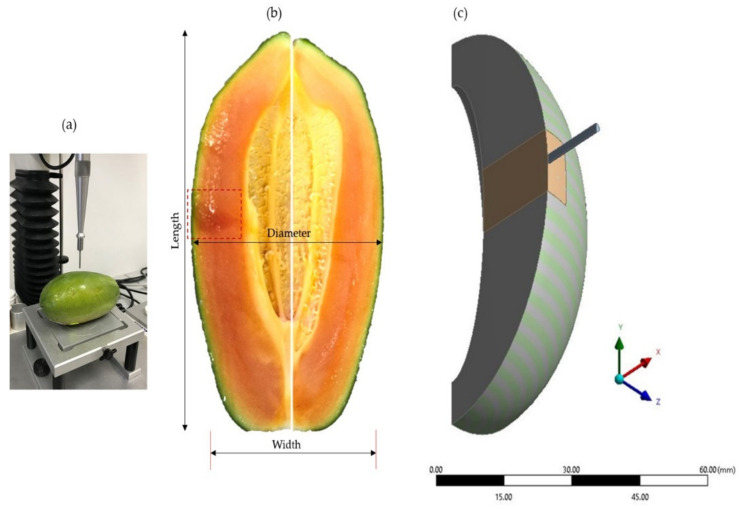
Picture of the actual puncture setup (**a**); the geometrical properties of papaya as viewed from the half-cut-top view (**b**); and the respective geometrical modelling (**c**).

A total of 10 fruits for each storage replication were subjected to puncture test, by using the similar universal testing machine (Instron 5543, Instron Corp., Norwood, USA). The needle-like flat-end probes of 2 mm (P/2) were attached to the load cell to penetrate into the intact skin and flesh tissues. Each fruit was positioned centrally on the platform as the probe moved down at the point of maximum diameter of the fruit. (Figure 2). The fruit was punctured up to 10 mm of deformation with a probe speed of 1.5 mm/s, 2 mm/s, and 2.5 mm/s. Datapoint acquisition was set at 200 points per second (pps) to generate the force-deformation profile. The rupture deformation and the bioyield force was defined by observing the probe tip completely enter into the fruit tissues.

### 2.4. Finite Element Analysis

#### 2.4.1. Geometrical Modelling

For each storage replication, two fruits were used for the assessment of geometrical characteristics. The fruit was cut using a sharp knife, along with a blossom end, and the coloured image of the section was captured using the digital camera (QICAM Color Fast 1394, QImaging, Surrey, BC, Canada) with a zoom lens (F5.6 and focal length of 18 mm). The longitudinal axes containing the major dimensions of length, width, and diameter were measured. A total of 15 points were randomly chosen for the measurement of skin and the flesh thickness, respectively. Over storage, there was no significant difference in the length, width, and diameter of 72.53 ± 2.97 mm, 83.07 ± 3.07 mm, 83.09 ± 2.75 mm, and 0.87 ± 0.03 g/cm^3^, respectively. Additionally, no significant variation of skin (1.01 ± 0.02 mm) and flesh thickness (3 ± 0.05 mm) was observed. Therefore, a multiscale model consisting of skin and flesh was proposed, by considering the mean values of the measurements of each geometrical property [21].

Figure 2 illustrates the steps involved in developing the geometrical model of the puncture test. Geometrical frames of skin and flesh were created using the “polyline” and “spline” command. Using the “extrude”, “revolve”, and “translate” commands, the solid papaya model was created. A “SurfFromFaces” command was used to define the skin thickness of 1 mm. The proposed model aimed to observe the rupture in the sample, due to the separation of the adjacent elements under the probe tip. Therefore, only the cross-section (15 × 20 mm) instead of the entire geometrical was considered to define the contact area, while the rest of the solid bodies of skin and flesh were suppressed. The puncture probe was modelled into a 3D rigid body. For viewing purposes, the “Slice” function was used to illustrate half of the contact area model. All geometries were modelled using the ANSYS^®^ Design Modeller (ANSYS Incorporation, Canonsburg, PA, USA).

#### 2.4.2. Contact Modelling, Meshing, and Boundary Condition

The FE model was simulated and analysed by using the ANSYS Workbench platform (ANSYS Incorporation, Canonsburg, PA, USA). For modelling the puncture, body interaction was selected to model the eroding contact between the probe, skin, and flesh bodies. The exterior surface of the skin sample was defined as a contact area with the rigid probe. The advanced settings of eroding contact options of plane symmetry, erosion interior node, and solid element treatment were set under program-controlled.

The edge sizing method was applied for the edge bias control between the elements of the cross-sectional and the suppressed bodies [22]. The linear-first-order of tetrahedral elements of solids and shells were utilized to model the element structures of flesh and skin, respectively. To ensure perfectly tied surfaces, the node positions of skin and flesh samples needed to be compatible [13]. Plus, patch-conforming and patch-independent algorithms were used for the element control. The effects of mesh sizes and shapes on the resulted stress were checked to decide on mesh density [23]. Using the inverse analysis, the pre-determined element sizes and shapes were modified until the simulation results agreed with the experimental results [24]. Subsequently, the 1 mm tetrahedral element was opted for to sufficiently represent the sample of papaya. The developed model had a total of 4557 nodes and 18,066 elements.

**Figure 3:**
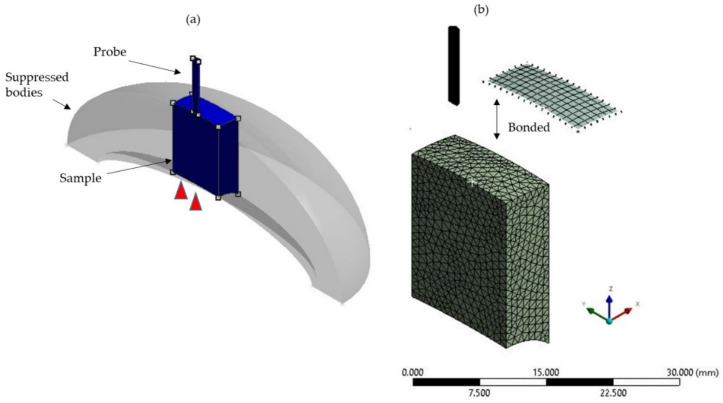
A 3D-finite element-puncture model: (**a**) sample model with a probe fixed at rigid part and a fixed support constraint at the bottom part; (**b**) meshed model with the tetrahedral elements.

A displacement boundary condition was applied to the reference point of the probe, so that the probe could only move down. At the loading point, the different insertion velocities of 10 mm/s, 20 mm/s, and 30 mm/s were applied. The sample was set to deform in the z-direction, in spite of the fixed x-direction and y-direction on the boundaries. A fixed support boundary condition on the bottom-face of the sample was applied to constraint all its degree of freedoms. Figure 3 illustrates the assembly of the FE-puncture model, which includes the sample and the probe. In addition, the effect of temperature was neglected during the simulation. Besides, the analysis-setting function in ANSYS also included the hourglass control, viscous damping, reduced integration, distortion control, and erosion dynamic mesh, which were used to maintain the model accuracy [15].

#### 2.4.3. Validation of FE Model

For the calibration of the FE model, repetitive simulations of the puncture experiment were performed based on the measured values of density, elastic modulus, yield stress, and tangent modulus and Poisson’s ratio of flesh and skin samples. Each probe was characterized as stainless steel with Young’s modulus of 20,000 MPa, with a density of 7850 kg/m^3^, in all simulation trials. The validation of the FE model was done by comparing the bioyield force and rupture deformation values obtained from the simulation and the actual puncture test.

### 2.5. Statistical Analysis

Data on the physical and mechanical properties of Exotica papayas at different storage days were analysed using the analysis of variance (ANOVA). The multi-comparison of each group’s means was then compared by Tukey’s test at *p* ≤ 0.05 significant level. To validate the proposed FE models, the relative error was calculated by comparing the simulation and experimental results by the following equation:

(3) $$R_{E}=\left|\frac{X_{E}\;\pm -X_{F}}{X_{E}}\right|\times 100\%$$

where $R_{E}$ is the relative error $X_{E}$ and $X_{F}$ is the data measured from the experimental and simulation procedure. The relation between simulation and puncture results was done by performing linear regression analysis. Statistical analysis was carried out using Minitab ^®^ 19.2 (Minitab Pty Ltd., Sydney, Australia).

## 3. Results and Discussion

### 3.1. Material Models

According to the ranges of the measurement values of the density, elastic modulus, yield stress, and tangent modulus and Poisson’s ratio of skin and flesh samples, the levels of minimum, average, and maximum values were listed in Table 1. Significant differences were observed in all measured properties between storage groups at *p* ≤ 0.05 significant level. Overall, all fruit properties were decreased with storage.

Determination of the constitutive model is vital for the success of FE simulation. From force-deformation curve data, the obtained elastic constant indicated a linear force-deformation curve, and the values of failure stress satisfy the bioyield point, which indicates the beginning of rupture [15]. The tangent modulus also related to the deformation behaviour, which was associated with the slope of the force-deformation curve. Hence, in this study, based on the inverse analysis and optimization method (data not shown), the elastic-perfectly plastic with linear hardening model was selected due to robustness and more accurate computation. It should be noted that the approach here assumes the elastic-plastic behaviour for both skin and flesh of papaya.

### 3.2. Puncture Mechanics

**Figure 4:**
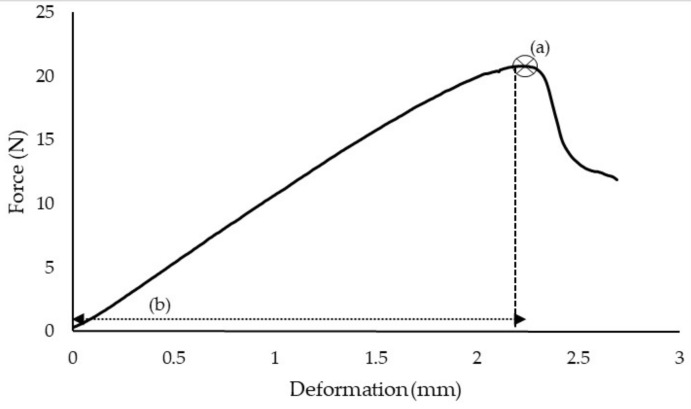
A typical force-deformation curve obtained from the puncture test: (**a**) bioyield point; (**b**) the corresponding deformation at the bioyield point.

Based on the typical force-deformation curve obtained from the puncture test, in the beginning, the linear deformation was governed by the applied force as the probe started to touch the fruit skin (Figure 4). At this point, deformation could be assumed to be recovered within the linear region. The skin tissues were assumed to begin to rupture at the bioyield point [25]. A distinct crack appeared after the rupture before the probe began to penetrate deeper into the flesh tissue. The fluctuation in force after the bioyield point of the force-deformation curve suggested that less puncture energy was required as the skin tissues had moved with respect to the motion of flesh tissues. The ongoing puncture made by the probe tip passed further, but there was no obvious increase or decrease in force towards the end of penetration. At this point, the contact area over the fruit tissues and the probe tip was assumed to remain constant. During the puncture, the force was assumed to be at the lowest at the centre of the probe and the highest at its perimeter [11,26]. Significant differences were observed in bioyield force and deformation under the influence of different storage intervals and puncture velocities at *p* ≤ 0.05 significant level.

### 3.3. Validation of Finite Element Model

**Figure 5:**
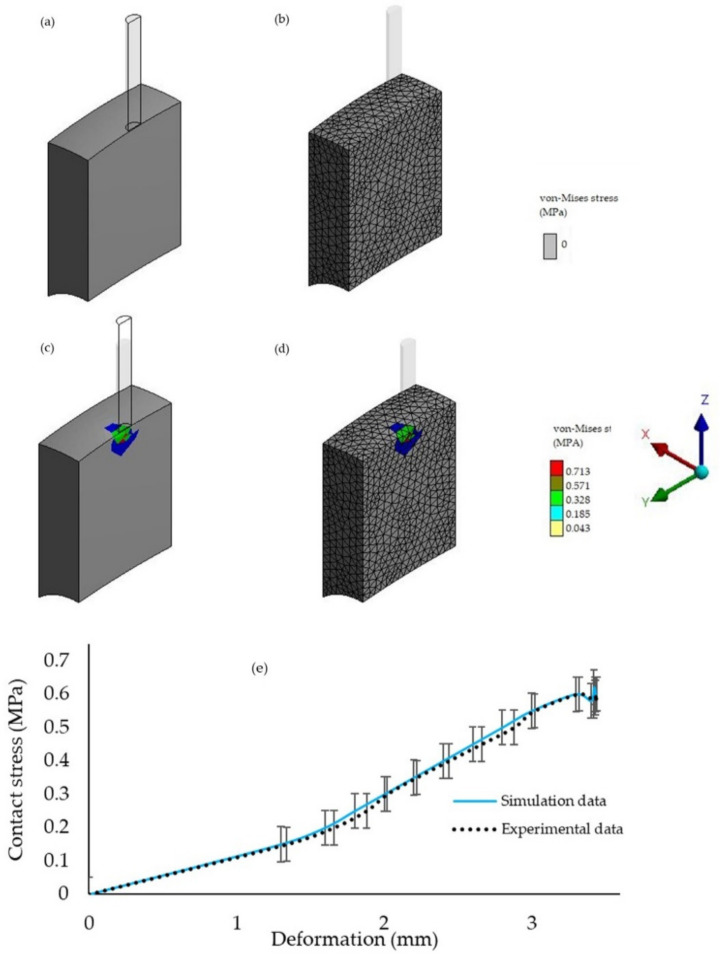
Deformation and stress waves at different puncture times: (**a**,**b**) shows the deformation situation and the corresponding mesh structures at 0.1 s; (**c**,**d**) shows the deformation situation and the corresponding mesh structures at 2.2 s; (**e**) stress-deformation curves based on the simulation and experimental data.

Figure 5a–d shows the typical pattern of deformations and stress waves of the FE model of the sample after the simulated puncture with a rigid needle-like probe under the prescribed insertion velocity. The von Mises criterion was used to estimate the total contact stress, which was regarded as the sum of shear and principal stress components throughout [27]. In general, the simulation model was able to discriminate the differences in the apparent puncture stress being experienced by the samples at the different puncture times. It was obvious that the stress was distributed from the tip of the rigid body and created a zone of higher stress right under the probe tip surface. During the puncture, a series of stress waves was created by the impact of the probe propagating into the sample. These stress waves moved inward more rapidly and notably, the sample did not begin to deform until the stress waves encountered the edges of the sample. The probe then gradually penetrated until the fruit reached up to the maximum of insertion depth at the end of the simulation time. Figure 5e shows the comparison between stress-deformation curves of simulated and experimental data. The contact stress increased from 0 to 0.71 MPa when the sample in the model was punctured at 1.5 mm/s.

The sensitivity of the FE model was further evaluated based on the ranges of the measurement values listed in Table 1. The results indicated that there were significant differences in contact stress and deformation responses. Good correlations were observed between bioyield force, deformation, elastic modulus, yield stress, and tangent modulus. During the puncture simulation, the contact force decreased by 52% (ranging from 19 to 9 N) and the deformation was decreased by more than 27% (ranging from 1.45 to 2.2 mm). Overall, the results suggested that there was a clear influence of these properties for the firmer fruit resulting in a stiffer response during yielding [28,29]. The results were in agreement with the earlier research conducted by [21] to determine the mechanical responses in tomatoes under quasi-static compression. Although the simulated results agreed well with most of the experimental values, the effects of different densities and Poisson’s ratios were predicted to be insignificant.

Table 2 shows the comparisons of bioyield force and deformation predicted by FE model and the actual puncture tests, based on the ranges of measurement values of elastic modulus, yield stress, tangent modulus, Poisson’s ratio, and density. The simulation and experimental curves were in agreement in all levels of puncture velocities, together with the 95% confident intervals (*p* ≤ 0.05) of the means for all measurements. The validation results confirmed that FE models are able to predict the bioyield force with a relative error of less than 15%. Although, FE model suggested the lower tolerance of simulated deformation. This could be related to the fruit model having unproportioned behaviour from the actual puncture setup or could have resulted from certain geometric configurations of the solid models [30]. Therefore, the simulation might be improved by establishing a more realistic 3D model of papaya and a puncture probe.

Overall, the bioyield force and deformation in papaya upon puncture were related to the stiffness state of papaya and the impact velocity. The authors of [31] reported that the differences in force values on the same group being punctured at different velocities were directly proportional to fruit firmness. The relationships between the difference in bioyield force and deformation at each velocity level followed the following quadratic polynomial equations:

(4) $$\Delta \mathrm{BF}=-2\;v^{2}+1.5\;v,\;\;R^{2}=0.92\;$$

(5) $$\Delta D=0.035\;v^{2}+0.12v,\;\;R^{2}=0.58\;$$

where the ΔBF is the difference of the bioyield force and ΔD is the difference of deformation at the given puncture velocity. The FE model was able to accurately predict the decrease in bioyield force over a different time, subject to the constant value of puncture velocity.

## 4. Conclusions

A novel, realistic, time-dependent, nonlinear, puncture test by means of FE simulation study was introduced. This study simulated the time-dependent puncture responses in papaya with a view of comparing the different constitutive-material models. To pave the way for further development of the FE model, this study concludes with the following:

- A 3D geometrical model of the papaya sample and puncture test system was developed. The boundary conditions of the load direction, displacement, and impact velocity were defined to mimic the actual puncture test.
- The 2 mm stainless-steel flat indenter probe progressed into the fruit tissues at different impact velocities of 1.5 mm/s, 2 mm/s, and 2.5 mm/s. The discrepancy of simulation results was related to the different values of Young’s modulus, failure stress, and tangent modulus of papayas.
- Although a useful tool, one major drawback is that it is limited to the linear elastic-plastic response of the sample.

In conclusion, the FE model developed in this study has the potential to serve as a simple and reliable prediction method to predict the yielding in papayas upon puncture loading. The simulation and modelling of the puncture by means of FE method can be beneficial to predict wounding in papaya due, for instance, to the effect of cutting, which can occur during pre- and post-harvest processing. The simulation data also can be useful during designing and prototyping related agricultural machinery and instruments, before building the actual versions. Further complexities can be built on to address the extensive range of puncture scenarios that could be possible in the future.

## Figures and Tables

**Table 1:** Levels of mechanical properties for the input of material model.

Material	Elastic Modulus (MPa)	Yield Stress (MPa)	Tangent Modulus (MPa)	Poisson’s Ratio	Density (kg/m^3^)
Skin	2, 6, 15	0.5, 2.5, 4	0.02, 0.03, 0.04	0.43, 0.44, 0.45	1000, 1100, 1200
Flesh	0.5, 2, 4	0.05, 1, 1.8	0.002, 0.003, 0.004	0.43, 0.44, 0.45	1000, 1100, 1200

**Table 2:** Some predicted values of bioyield force and deformation predicted in finite element model, indicated by the relative error.

Storage Interval (d)	Velocity (mm/s)	Level	Bioyield Force (N)		Error (%)	Deformation		Error (%)
			Measured	Simulated		Measured	Simulated	
0	1.5	1	17.81 ± 0.64	17.30 ± 0.091	2.86	1.5 ± 0.29	2 ± 0.15	33.33
	2	2	17.87 ± 2.22	17.35 ± 0.11	2.91	1.67 ± 0.27	2.15 ± 0.16	28.74
	2.5	3	18.36 ± 0.08	17.57 ± 0.14	4.30	1.8 ± 0.12	2.2 ± 0.15	22.22
4	1.5	1	14.87 ± 0.87	14.76 ± 0.27	0.74	1.21 ± 0.07	2 ± 0.20	65.28
	2	2	15.31 ± 2.11	15.81 ± 0.19	3.26	1.35 ± 0.09	2.1 ± 0.21	55.55
	2.5	3	16.42 ± 0.91	16.81 ± 0.22	2.37	1.45 ± 0.17	2.35 ± 0.29	62.06
7	1.5	1	13.20 ± 2.13	11.29 ± 0.19	14.46	1.22 ± 0.16	1.8 ± 0.15	47.54
	2	2	13.43 ± 0.89	12.22 ± 0.20	9.00	1.38 ± 0.05	1.85 ± 0.18	34.05
	2.5	3	13.55 ± 1.33	14.29 ± 0.20	5.46	1.43 ± 0.44	1.9 ± 0.16	32.86

Data represents the mean values and the replicates (± standard error).
